# Pollination Mechanisms are Driving Orchid Distribution in Space

**DOI:** 10.1038/s41598-020-57871-5

**Published:** 2020-01-21

**Authors:** Zuzana Štípková, Spyros Tsiftsis, Pavel Kindlmann

**Affiliations:** 1 Global Change Research Institute, Academy of Sciences of the Czech Republic, České Budějovice, 37001 Czech Republic; 2 Institute for Environmental Studies, Faculty of Science, Charles University, Prague, 12801 Czech Republic; 3 Department of Forestry and Natural Environment, International Hellenic University, Drama, 66100 Greece

**Keywords:** Ecology, Ecology, Environmental sciences

## Abstract

Understanding the abundance and distribution patterns of species at large spatial scales is one of the goals of biogeography and macroecology, as it helps researchers and authorities in designing conservation measures for endangered species. Orchids, one of the most endangered groups of plants, have a complicated system of pollination mechanisms. Their survival strongly depends on pollination success, which then determines their presence and distribution in space. Here we concentrate on how pollination mechanisms (presence/absence of nectar) are associated with orchid species density and mean niche breadth along an altitudinal gradient in six different phytogeographical regions in the Czech Republic. We found differences between these regions in terms of orchid species numbers and density. The trend (hump-shaped curve) in species density of nectarless and nectariferous orchids were very similar in all phytogeographical regions, peaking between 300–900 m. The trend strongly depends on habitat cover and pollinator availability. In general, the most specialist species of orchids were found from low to middle altitudes. The association of altitude with the richness of orchid flora is much stronger than that with the biogeography. Climate change is a factor that should not be neglected, as it may affect the presence/absence of many species in the future.

## Introduction

Orchids are disappearing worldwide^1^, mostly due to habitat loss, but other factors (e.g. climate change and resulting shifts in species distributions) are also expected to increase in importance during the 21st century^2,3^. Thus, it is worrying that we still do not know the optimal requirements for the majority of the ~30,000 species of orchids existing on Earth^4^. There are only a few studies on the factors that determine orchid presence/absence and distribution in space and most of them include only one or a few species and/or a limited part of the distribution of the species studied, e.g.^5,6^. As a result, we lack critical information necessary for the conservation of Orchidaceae, especially the species that are known to be threatened or endangered. Therefore, understanding the abundance and distribution patterns of species at large spatial scales is one of the key goals of biogeography and macroecology^7–9^. The lack of knowledge about orchid ecology and distribution also negatively affects our ability to identify sites that are worth protecting. We also lack the knowledge needed to develop management plans for orchids under current or future scenarios of habitat loss and climate change.

Recently, a step was taken in this direction by Tsiftsis *et al*.^9^, who explored the associations between orchid species density, mean niche breadth and mean distribution on the one hand and selected predictors on the other hand, using regression techniques for all orchids, and then for those with different root systems. Each root system is thought to represent a particular strategy for underground storage of resources and resource acquisition. In this sense, Tsiftsis *et al*.^9^ distinguished three categories of orchid species, based on the morphology of their root system: (i) rhizomatous orchids (the most primitive), (ii) “intermediate orchids” (those with attenuated – palmate, fusiform or stoloniferous tubers—in evolutionary history intermediate between rhizomatous and tuberous orchids) and (iii) tuberous orchids (those with spheroid tubers – the most advanced). Tsiftsis *et al*.^9^ then show that species density for the three below ground strategies is significantly associated with the predictors, whereas their mean niche breadth and mean distribution largely are associated with their evolutionary history represented by the corresponding root system.

Besides the root systems, however, there is another life history trait that may play a significant role in determining orchid presence/absence and distribution in space: pollination. Survival of an orchid population or even a species may strongly depend on pollination and subsequent seed production^10^. As specialized pollination systems may be particularly vulnerable to anthropogenic landscape modification^11–13^, the type of pollination system may strongly affect species survival.

Most plants that are pollinated by animals produce and offer rewards to attract pollinators to visit their flowers (nectariferous species^14^). Nectar is considered the most common floral reward^15,16^ and can influence several aspects of pollinator behaviour^16^. However, some plants attract pollinators although they do not offer them any reward in their flowers (nectarless species^17,18^). The nectarless strategy has evolved in many plant families, but most of nectarless species are orchids^19,20^. Generally, orchids are characterized by a diversity and specificity of pollination mechanisms, which may involve food-foraging, territorial defence, pseudoantagonism, rendezvous attraction, brood-site and shelter imitation, sexual response, or habitat-selection behaviours of their pollinators^20–28^. Nevertheless, it is convenient to divide orchids simply into nectariferous and nectarless species.

In general, plants of nectariferous species are visited more frequently than nectarless plants^29–31^. Pollinators also visit more flowers per inflorescence of nectariferous than in nectarless species^16,31,32^. Nectariferous species are less pollinator-specific than deceptive species, among which the most pollinator-specific are sexually deceptive species^32,33^. As much as 60–70% of orchids have a single pollinator species^26^. This specialization for a single or a few pollinators^23,33^ makes orchids vulnerable to fluctuations in pollinator abundance. Nectariferous orchids are better competitors for pollinators than nectarless orchids^30^. All this has consequences for fruit production and therefore fitness of the plants. As a result, nectariferous species have a higher fruit set than nectarless ones^26,29,31,33^ in all geographical areas^29^ due to pollination limitation^26,29^.

All the above affect the altitudinal and spatial distribution of orchids, as well as a range of ecological conditions. For example, on La Reunion Island, Jacquemyn *et al*.^34^ report that animal-pollinated orchids are more abundant at lower altitudes, while at high altitudes orchids tended to be auto-pollinated and cleistogamous. In Switzerland, the relationship between altitude and frequency of orchids of different reward strategies indicates a significant decrease in the occurrence of generalized nectarless species of orchids with increase in altitude^30^.

In addition to the pollination strategy, pollinator abundance can also affect fruit set in orchids. Pollinator abundance is influenced by climate (temperature, seasonality) in a given area, which in turn is strongly determined by altitude^35,36^. Although hypotheses testing association of species richness and niche breadth with altitude are frequently referred to in the literature, e.g.^37–41^ and so on, there are only a few such studies, like that of Tsiftsis *et al*.^9^, on orchids. None of these studies distinguished between pollination strategies (nectariferous/nectarless).

There are six different phytogeographical regions in the Czech Republic. They differ in altitude, but also in the spatial distribution of different habitats and their geological substrate^42^ as well as in the intensity of human activities in the past. This may also affect the distribution of orchids. Therefore, it is necessary to analyse each of the phytogeographical regions separately, instead of only one or in all of them together.

Here we test, whether there are differences between trends in species density and mean niche breadth between nectariferous and nectarless species, along an altitudinal gradient. We perform these analyses for the six phytogeographical regions in the Czech Republic.

## Results

**Figure 1:**
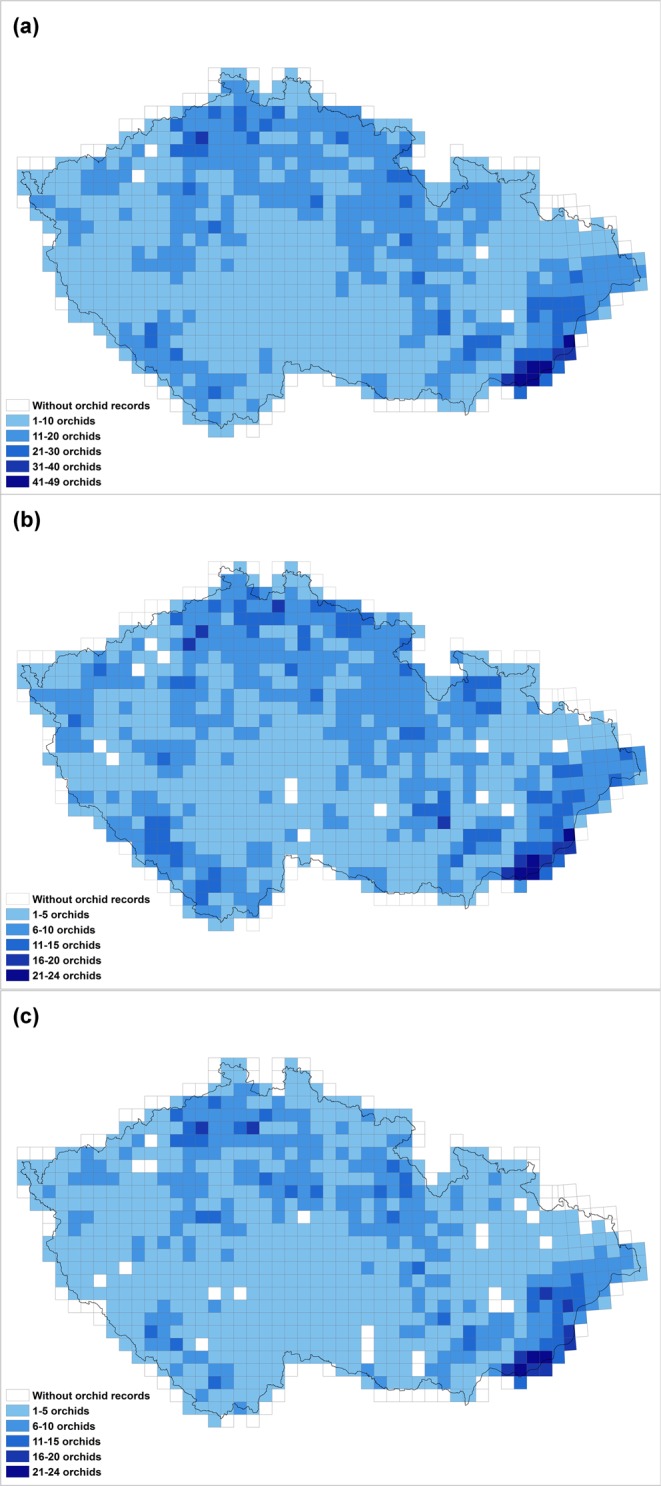
Maps showing the distribution of the density orchid species in the Czech Republic: (**a)** total number of orchid taxa; (**b)** nectariferous orchids; (**c)** nectarless orchids. The maps were generated in ArgGis (version 10.1, www.esri.com).

For our analyses, we used 68 out of 70 taxa, except hybrids, referred in Danihelka *et al*.^43^ and *Dactylorhiza fuchsii* subsp. *carpatica* (the reason was explained above). In the Czech Republic, there are 37 nectariferous and 32 nectarless orchid taxa (Supplementary Table S1) recorded in 858 out of the 916 grid cells (Fig. 1a). The most species-rich areas in the Czech Republic are in the south-eastern part of the country (Bílé Karpaty and Beskydy Nature Conservation Area), in the Šumava National Park in the south western part, mountainous areas in the north on the borders with other countries (e.g. National Park of Krkonoše and České Švýcarsko, Jeseníky Nature Conservation Area) and also some smaller inland areas.

Nectariferous orchids, recorded in 841 grid cells (Fig. 1b) were more widely distributed in the country than the nectarless orchids, which were recorded only in 822 grid cells (Fig. 1c). Even in terms of number of records (not presented here), there are many more records of nectariferous orchids in each of the six phytogeographical areas, except for the thermophyticum where the difference between these two groups is small, but again in favour of nectariferous orchids (thermophyticum: 180 records of nectarless orchid taxa vs. 187 records of nectariferous orchids; mesophyticum: 320 nectarless orchids vs. 383 nectariferous orchids; oreophyticum: 156 nectarless orchid records vs. 223 nectariferous orchid records).

In general, the patterns for both groups are similar throughout the Czech Republic, with the greatest number of species in both groups recorded in the south-eastern part of the country. Despite this similarity, the Mann-Whitney U test indicates there is a significantly higher number of nectariferous than nectarless orchids in this country (P < 0.001).

On the basis of the composition and distribution of orchids in the six phytogeographical areas in the Czech Republic, the hierarchical cluster analysis indicates three statistically significant clusters (P < 0.01, Supplementary Fig. S1). Despite the differences in the spatial distribution of these clusters in this country, the clustering was based on the climatic conditions, which characterize these areas (e.g. Bohemian thermophyticum clustered with Pannonian thermophyticum) and are associated with the distribution of the orchids.

The analysis of the differences in the number of grid cells, where each orchid is recorded in the whole area of the Czech Republic, revealed that nectariferous orchids are distributed in higher numbers in the 10×10 km UTM grid cells (more widely distributed) compared to the nectarless taxa, but the differences, according to the Mann-Whitney U test, are non-significant (P = 0.782). The same trend was also found when the comparison was based on grid cells with 30-sec spatial resolution (P = 0.420). The trends in the distributions of the two groups of orchids in the phytogeographical areas in the Czech Republic are presented in Table S2. Nectariferous orchids are more broadly distributed than nectarless orchids in 4 of the 6 phytogeographical areas. However, the trends are statistically significant for only two of them. The Mann-Whitney U test revealed that at lower altitudes, characterized as “thermophyticum”, nectarless orchids are more broadly distributed, despite the non-significant results.

**Figure 2:**
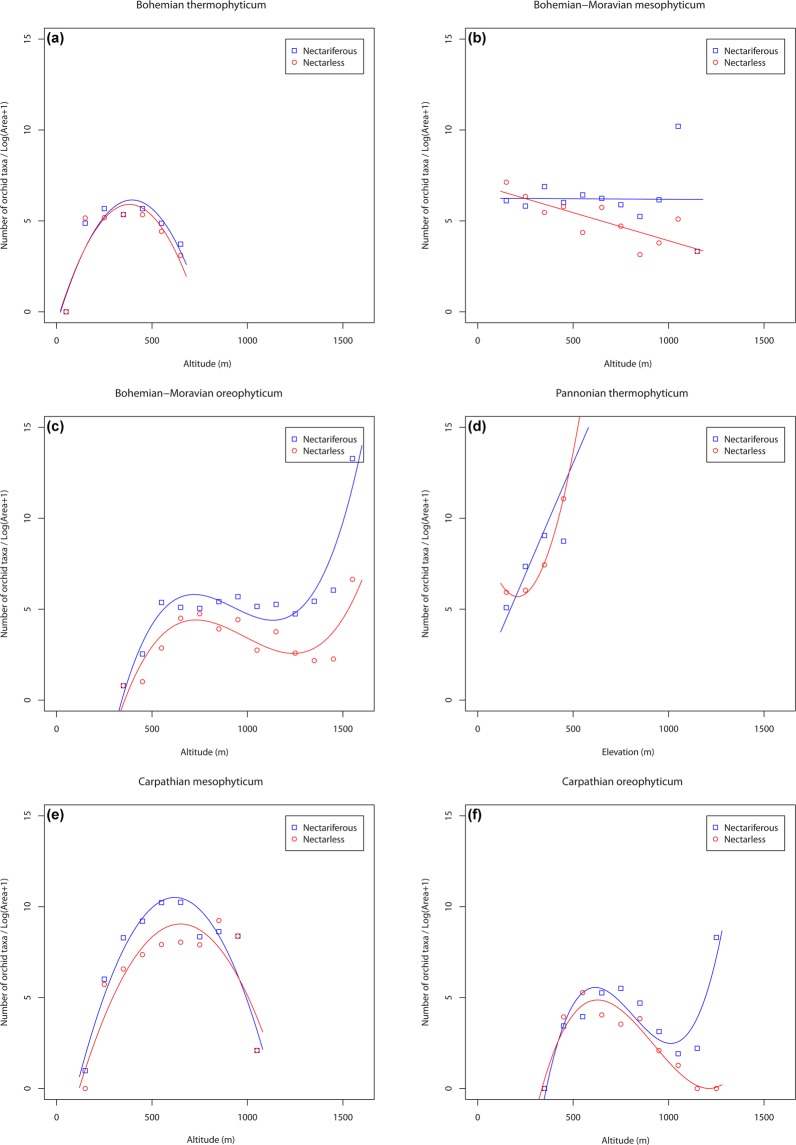
The trends in different pollination mechanisms of orchid taxa in: (**a)** Bohemian thermophyticum, (**b)** Bohemian-Moravian mesophyticum, (**c)** Bohemian-Moravian oreophyticum, (**d)** Pannonian thermophyticum, (**e)** Carpathian mesophyticum and (**f)** Carpathian oreophyticum. Blue colour represents nectariferous species, while red colour represents nectarless species. Squares and circles show the number of orchid taxa in each orchid group (nectariferous and nectarless) in particular altitudinal interval (intervals were set to 100-m, see Methods for more information).

The trends in orchid species density along the altitudinal gradient are shown in Fig. 2. The trends for the two groups studied are not very different. Specifically, in the Bohemian thermophyticum (Fig. 2a) the distribution of both nectariferous and nectarless orchid groups have a hump-shaped patterns with their number increasing up to c. 350 m a.s.l. The number of nectariferous species of orchids then decreases markedly, whereas that of nectarless species decreases only slightly with increase in altitude. The total number is the same for both groups in this region. Similar patterns were also recorded in the phytogeographical area of the Carpathian mesophyticum (Fig. 2e), but in this case the altitudinal range, over which orchids were recorded is much greater than in the Bohemian thermophyticum and the maximum number of orchids was recorded at c. 600 m a.s.l.

Unlike these two phytogeographical areas, where the species show a hump-shaped trend, in the Bohemian-Moravian mesophyticum (Fig. 2b), the nectarless orchids show a monotonically decreasing, trend, whereas the numbers of nectariferous orchids do not change along the altitudinal gradient. In the Bohemian-Moravian oreophyticum, the curves have the same shape for both pollination groups, but there are slightly more nectariferous species in this region (Fig. 2c). Both groups peak at about 750 m a.s.l., then their numbers decrease slightly at 1,200 m a.s.l. in the case of nectariferous species, and at 1,300 m a.s.l. in case of nectarless species, respectively. After that, the number of both groups increases again up to the highest altitudes.

The Pannonian thermophyticum area is characterised by a sharp increase in numbers of species of both nectariferous and nectarless orchids (Fig. 2d), whereas in the Carpathian oreophyticum (Fig. 2f), the species density of both nectariferous and nectarless species increases up to 600 m a.s.l., then the number of nectariferous species decreases up to c. 1,000 m a.s.l. and then increases quickly up to the highest altitudes in this region. In contrast, the number of nectarless species decreases with increasing altitude with no sharp increase at the highest altitudes.

Most of the correlations between orchid species density and altitude in the six phytogeographical areas in the Czech Republic were statistically significant (P < 0.05; Supplementary Table S3), and their predictive power was very high (R^2^ = 62–99%). In contrast, the only non-significant association was that of the number of nectariferous species along the altitudinal gradient in the Bohemian-Moravian mesophyticum, the predictive power of which was very low (R^2^ = 11%). The low R^2^ value and the non-significant regression can be attributed to the large number of orchids (12 taxa) recorded in a small area between 1,000 and 1,100 m a.s.l. (14 grid cells), a value that was as an outlier in the regression analyses.

**Figure 3:**
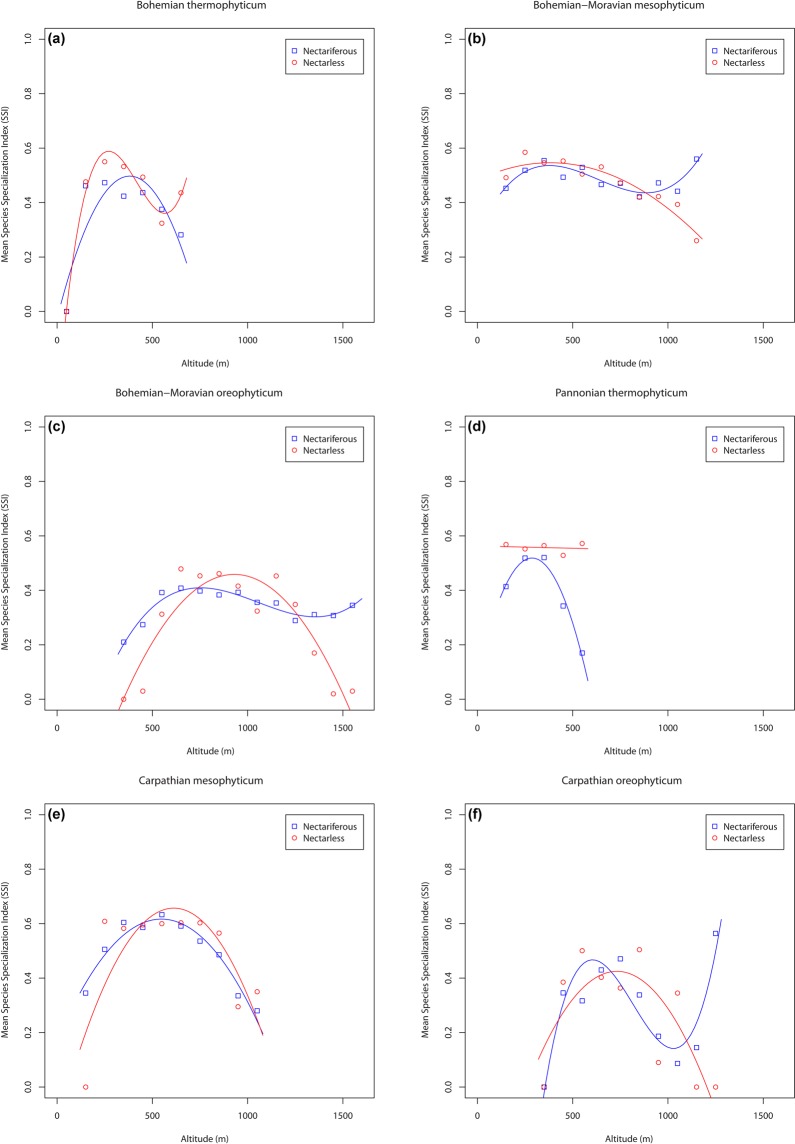
The specialization in pollination system (differences between nectariferous and nectarless orchid taxa) in: (**a)** Bohemian thermophyticum, (**b)** Bohemian-Moravian mesophyticum, (**c)** Bohemian-Moravian oreophyticum, (**d)** Pannonian thermophyticum, (**e)** Carpathian mesophyticum and (**f)** Carpathian oreophyticum. Blue colour represents nectariferous species, red colour represents nectarless species. Squares and circles show the mean species specialization index (SSI) in each orchid group (nectariferous and nectarless) in particular altitudinal interval (intervals were set to 100-m, see Methods for more information).

The relationship between mean niche breadth (represented by values of mean species specialization index - SSI) and altitude for each of the two orchid groups in the phytogeographical areas in the Czech Republic are presented in Fig. 3. This shows that the highest number of specialist nectarless species in the Bohemian thermophyticum occurs at around 260 m a.s.l. and increases again at altitudes of more than 600 m a.s.l. (Fig. 3a). The curve of nectariferous species is hump-shaped, peaking at an altitude of about 380 m a.s.l. However, the results for nectariferous species in this area are not significant.

In the Bohemian-Moravian mesophyticum (Fig. 3b), nectarless species tend to have a narrower niche breadth (more specialists are present) at high altitude than those recorded at lower altitudes. The SSI values of nectariferous species are rather stable along the altitudinal gradient in this floristic region and only at high altitudes (>1,000 m) does the mean SSI increase slightly.

Both nectariferous and nectarless orchids occurring at low altitudes in the Bohemian-Moravian oreophyticum (Fig. 3c) tend to have broad niche breadths. At high altitudes, nectariferous species tend to be more specialized (have a narrower niche breadth) – a trend that continues up to the high altitude areas, whereas for nectarless orchids, the narrowest niche breadth is recorded at an altitude of c. 900-1,000 m a.s.l. and in areas above 1,000 m a.s.l. orchids again characteristically have broad niches (generalists).

Although the results for nectarless and nectariferous species are not significant in the Pannonian thermophyticum phytogeographical area (Supplementary Table S4), there are different trends in the two groups of orchids. It is clear from Fig. 3d that the mean SSI of the nectarless orchids does not change with altitude, whereas that of nectariferous orchids has a unimodal trend.

In the Carpathian mesophyticum, the results for both groups (nectarless and nectariferous species) are very similar with respect to the shape of the regression line (Fig. 3e). Specifically, both orchid groups show a unimodal trend with a peak at c. 500–600 m a.s.l.

In Carpathian oreophyticum, nectarless species tend to have narrower niche breadths at c. 750 m a.s.l. compared to those that are found both at low and high altitudes (Fig. 3f). Specialist nectariferous species tend to occur at altitudes of about 620 m a.s.l. and the index of specialisation increases at altitudes above 1,100 m. At the lowest altitudes and at about 1,000 m a.s.l. in this region, nectariferous species are not so specialized, as they occur in a wide range of environmental conditions.

For the mesophyticum and oreophyticum phytogeographical areas, all the associations are statistically significant (P < 0.05; Supplementary Table S4) and the respective values of the predictive power are also high (R^2^ = 51–93%). In contrast, non-significant trends were detected in both thermophyticum phytogeographical areas (except for the nectarless orchids in Bohemian thermophyticum) where the predictive power is also low (R^2^ = 31–59%).

## Discussion

### Orchid species density along the altitudinal gradient

**Figure 4:**
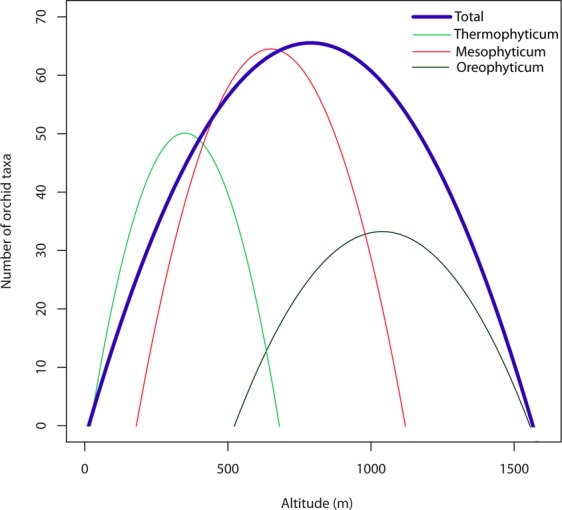
Orchid species density along the altitudinal gradient within each of the phytogeographical regions in the Czech Republic.

Earlier studies demonstrate that species richness can be described by a hump-shaped curve with respect to altitude: it increases with altitude at low altitudes, is highest at mid-altitudes and above this it decreases with increasing altitude^34,44,45^. On Réunion Island, the highest species richness occurs between 400 and 800 m^34^. Although these studies were done in tropics, our results for a temperate climate also support the existence of a hump-shaped curve. In our case, the highest species density was recorded between 300 and 900 m. In addition, similar curves occur even within each of the phytogeographical regions in the Czech Republic. This is depicted in Fig. 4: in the lowest parts of the country (thermophyticum, up to 600 m a.s.l.), the highest number of orchid taxa is recorded around 300 m, in mesophyticum (mid-altitudes from 200 to 1,100 m a.s.l.) around 500 and in the highest parts of the country (oreophyticum; from 400 m a.s.l.) around 900 m.

The high species richness and density at mid-altitudes may be explained by the presence of a zone, where the altitudinal distributions of many orchid species overlap^34^. It makes sense, because lowland species may occur also at higher altitudes, while high-altitude species may occasionally be found at lower altitudes. However, at least in case of the Czech Republic, the lower number of orchid taxa recorded at both low and high altitudes in the six phytogeographical regions can also be attributed to the absence there of suitable orchid habitats (see Fig. 4).

### Nectarless vs. nectariferous species

In the Czech Republic, there are more nectariferous than nectarless species of orchids, which is consistent with Neiland & Wilcock^29^.

From Fig. 1, it is obvious that nectarless and nectariferous orchids are present almost in all of the grid cells and often both groups occur in one cell. It seems that this is not rare, as it has been also observed in other countries as well, e.g. in the Netherlands and Flanders, where nectarless and nectariferous species also have similar distributions^10^. One of the reasons may be that although nectarless species do not provide any reward to the pollinators, they benefit from the vicinity of nectariferous co-flowering plants that attract pollinators and in this way increase their local abundance – the “magnet species” hypothesis^46–48^, which considers also “magnet” species belonging to other plant families.

### Orchid species richness in different phytogeographical areas

In the cluster analysis (Supplementary Fig. S1) the phytogeographical areas were grouped together according to their altitudinal zone. This clearly demonstrates that the association of altitude with the composition of orchid flora in the Czech Republic is much stronger than that of biogeography. This differs from the patterns identified in other countries (e.g. Greece – see Tsiftsis *et al*.^9^), where different areas host different groups of orchid taxa. This may be due to differences in the distribution and height of mountain ranges in the Czech Republic and Greece. The vast majority of Czech mountains are mainly at the borders with other countries, so orchid seeds are easily distributed between adjacent regions. Of course, mountains in the Czech Republic are not very high (only up to 1,600 m), unlike in Greece where there are lots of inland mountains that may not be easily crossed by orchid seed. A similar situation to that in Greece is, for example, occurs in Colombia. The high Andes cannot be easily crossed by many species, not only orchids, which results in each valley hosting a different flora and fauna^49,50^. Another explanation may be associated with the position of the Czech Republic. The country is not too wide from north to south, so latitude does not play a significant role, unlike the situation in Greece.

In contrast to the similar hump-shaped trends in orchid species density along the altitudinal gradient (Fig. 4), orchid species density strongly differed in the six phytogeographical regions, whereas the trends in species density of nectarless and nectariferous orchids in each of these regions are very similar. The only exception is the Bohemian-Moravian mesophyticum, where the dependence decreases for nectarless and is constant for nectariferous species.

The difference in the density of orchid species may be attributed to differences in habitat cover in each region, mainly at the highest altitudes in this country^51^. We hypothesize that this may be due to either.

(i) open areas having a higher diversity of bees and butterflies and hosting a higher number of plant species than shaded areas^52–56^
  or as reported by Eckerter *et al*.^57^
(ii) there is higher pollinator activity on flowers at sunny sites. This has two consequences: first, orchids growing in meadows are more likely to be pollinated, as insects are attracted by other meadow species (even belonging to different plant families) that grow nearby (so-called magnet species hypothesis – see above), second, orchids living in forests and other shady habitats have no other choice than to offer some kind of reward (nectar in this case) to attract foraging pollinators.

This may explain the difference between nectarless and nectariferous orchids in both oreophyticum phytogeographical regions. In the Carpathians, the highest altitudes are mostly covered by forests that favour more the distribution of nectariferous species. In the Bohemian-Moravian oreophyticum, the highest parts are also covered by thick forests, but there are more alpine meadows, marshlands and natural non-forest areas (mainly in Jeseníky, Šumava and Krkonoše mountains) that benefit nectarless species to a greater extent than in the Carpathian oreophyticum.

Another explanation may be the calcareous substrate that occurs to a much greater extent in the Pannonian region and Carpathians than in other parts where granite is more frequent. Of these two bedrocks, calcareous substrates are greatly preferred by orchids, whereas only a few species of orchids occur in area with granite bedrock^6,58,59^. On such type of bedrock, acidic soils are formed and it is what for example *Dactylorhiza viridis*, *Dactylorhiza maculata* or *Hammarbya paludosa* prefer^60^. Out of the first two species that occur in the Pannonian region, one is nectariferous (*D. viridis*) and the second is nectarless (*D. maculata*).

In addition, the distribution of pollinators may play a role in determining orchid species density and density differences in particular regions. Arroyo *et al*.^35^ report that the pollinator community at different altitudes differ and is a poorest at high altitudes.

### Relationship between mean niche breadth and altitude

The general distribution of each of the orchid groups studied is determined by the specific ecological requirements of the species in each group, the spatial distribution of the different habitats and the presence of pollinators and mycorrhizal fungi. These factors reflect the niche breadth of each species^10,61,62^. As a metric of niche breath, we used the species specialization index (SSI), calculated on the basis of the climatic conditions at the sites where orchids are recorded. Although species niche-breadth, in general, increases along altitudinal gradients^63^, a study of the Greek orchids indicate that these trends are mostly associated with their life forms^9^. Their findings are in accordance with our results. We have shown, in addition, that these trends can differ between areas.

We hypothesize that the differences in the trends in the six phytogeographical regions are the result of the area-specific distribution patterns of the orchids. In general, most specialist species of orchids occur from low to middle altitudes and there are only small differences in the distribution of nectarless and nectariferous species when different phytogeographical regions are considered. This was the case for both oreophyticum areas. In the Bohemian thermophyticum, most specialist species of nectariferous orchids, such as *Goodyera repens* and *Herminium monorchis*, occurred at middle altitudes, while for nectarless species (e.g. *Orchis pallens* or *Liparis loeselii*) most specialist species occurred at middle to high altitudes. In the case of the Carpathian oreophyticum, the distribution of specialists in both groups was the opposite.

This may be partly explained by the distribution of forest and grassland habitats. In both areas, there are deep shaded forests at the highest altitudes but there are much more natural non-forest areas in the Bohemian part, which is preferred by nectarless species^42,51,64^. However, high-altitude species in the Czech Republic in both Bohemian thermophyticum and Carpathian oreophyticum are nectariferous. This may support the hypothesis of Pellisier *et al*.^30^ that the frequency of nectarless species of orchids decreases with altitude, which implies that deception may be less profitable at high than to low altitudes. This, in turn, may be associated with the low number of pollinators at high altitudes^35^.

Several studies deal with orchid specialization in terms of the distribution of their pollinators^28,62,65–68^. Phillips *et al*.^28^ hypothesize that pollinator specialization in sexual deception^33^ makes orchids vulnerable to changes in pollinator abundance. They suggest that this is less likely in food-nectariferous or food-nectarless species, which attract a particular group of foraging insects.

Regarding the association of orchid species rarity and pollination strategy, Neiland & Wilcock^29^ found that nectarless species are more likely to be rare. There are similar results for orchids in south-western Australia^28^. However, Jacquemyn *et al*.^10^ state that orchid rarity is related more to habitat than to pollination strategy. We found that nectarless orchids were more restricted in their distribution (i.e., occurred in fewer grid cells; see the Mann-Whitney U test results in Supplementary Table S2) than nectariferous species. These trends, however, were mostly non-significant. We found that nectarless orchids were more common than nectariferous orchids only at low altitudes, characterized as “thermophyticum” (see Supplementary Table S2). This can be attributed to their species composition. About half of the orchid taxa recorded in areas characterized as “thermophyticum” belong to the genera *Dactylorhiza* (13 taxa) and *Ophrys* (3 taxa), which are mostly found in lowlands with extensive grassland communities (e.g. wet meadows, heaths, peat bogs, dry grasslands) or could be found in the past before the intensification of the agriculture, which began in 1948^69,70^. Nectariferous species were found to be more widely distributed in high altitude floristic areas (characterized as “mesophyticum” and “oreophyticum”), which may be attributed to the greater prevalence of forested habitats in these areas than in thermophyticum areas. In a forest, where visibility is often greatly limited, there is a greater need to provide a rewarding order to attract pollinators. However, most of these trends were non-significant and consequently they do not support that the rarest species are mostly found in the nectariferous or nectarless group. Moreover, we agree with Phillips *et al*.^28^ that the extinction of orchid species is not dependent on production of nectar but is significantly related to the survival of the habitats where these orchids occur^10^. The cause of orchid decline is believed to be more affected by landscape fragmentation and deterioration, which is closely connected to the loss of natural habitats^10,71^.

### Conservation implication

Studying orchid pollination mechanisms is crucial for their future survival, as orchids are dependent on seed production. Such studies can provide valuable information and conservation implications especially in the case of highly endangered species. Our results can help us to better understand orchid ecology and distribution, as well as orchid reproduction in relation to the likelihood of their extinction in the future. For example, as fitness of an orchid species is influenced by seed production, nectarless species should be more prone to local extinction than nectariferous species^10^.

In this study, we found that the majority of nectariferous species can be found in forest habitats, their reproductive success relies on nectar production and availability of their pollinators. There are lots of forests in the Czech Republic, but only a small part of them is somehow protected, whereas logging activities are still very common. It implies that also forest habitats, not only meadows, should be protected in terms of plant species preservation.

It is known that pollination is one of the most important issues in orchid life, but the production of nectar does not provide any guarantee against local extinction. We fully agree with Jacquemyn *et al*.^10^ that habitat loss and other threats associated with habitat fragmentation and deterioration are more important for orchid persistence. Despite the fact that our study uses only data from the Czech Republic, we believe that it is also applicable in other parts of Central Europe, as well as in other temperate regions.

Particular attention should be paid to the biology and requirements of the plant-pollinator relationships. Moreover, more long-term demographic studies including sufficient number of orchid species are needed to evaluate factors that affect the distribution patterns of nectarless and nectariferous species, especially in the environment changing due to human activities.

## Methods

**Figure 5:**
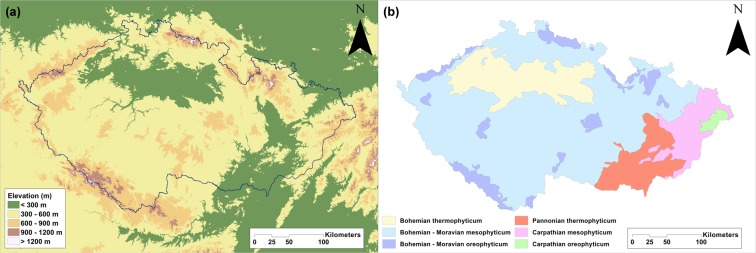
Maps of the Czech Republic showing: (**a)** the areas at different altitudes and (**b)** phytogeographical areas (taken and modified from Kaplan 2012). The maps were generated in ArgGis (version 10.1, www.esri.com).

Czech Republic (N 48°33′–51°03′, E 12°05′–18°51′, area 78 866 km^2^, altitude 115–1,602 m a.s.l.) was chosen for this study because of its position in central Europe and because its orchid flora is very well studied. Its average altitude (450 m a.s.l.) is slightly above the average for Europe: 290 m a.s.l. (Fig. 5a). It is covered mainly by highlands of moderate altitude (67% of the area is below 500 m a.s.l.). Higher mountains occur at the borders of this country, especially in the north and south.

The climate of the Czech Republic is typically temperate with cold, cloudy and humid winters and hot summers, although there are some regional and local differences due to shape of the relief that forms the complex topography in this area. For example, temperatures in the eastern part (so-called Moravian region) are a bit higher than in the rest of the country (Bohemian region). Because the Czech Republic is relatively small, temperature and precipitation are mostly affected by local vertical heterogeneity and altitude.

Czech Republic can be divided into three principal phytogeographical units (phytochoria) based on the dominant flora and vegetation that reflects specific regional geomorphological and climatic conditions – Thermophyticum, Mesophyticum and Oreophyticum^42^.

Thermophyticum includes warm areas with a thermophilous flora and vegetation that lies mainly in the lowlands. This region is characterized by the occurrence of basiphilous thermophilous oak and oak-hornbeam forests, dry scrub land and grasslands (*Festuco-Brometea* class), whereas peat bogs and beech forests are nearly absent. The remnants of softwood floodplain forests, loess deposits, calcareous fens, as well as local saltmarshes and saline meadows, can be found there.

Mesophyticum is the basic region with flora and vegetation typical for the Czech Republic and the Central European temperate zone. It is located in the foothills or lower slopes of mountains (sub-mountain belt). The potential natural vegetation in this region consists mainly of various types of mesic beech or hornbeam forests, meadows and grasslands, typically with *Arrhenatherum elatius, Molinia caerulea* and *Bromus erectus*, herbaceous forest edges and some specific communities, such as the vegetation growing on the exposed bottom of fishponds.

The last phytogeographical region, the Oreophyticum, is cold with a mountain flora and vegetation, corresponding to the forests of the boreal zone, smaller areas above the timberline similar to habitats in the arctic zone are also present here. Typical vegetation comprises mainly coniferous forests or mixed forests with a high abundance of conifers. Natural subalpine and alpine grasslands (above timberline) occur only at the highest altitudes, where montane and sub-boreal species can be found^42,64^. Each of these regions is further divided into two provinces.

As a result, in the Czech Republic, there are the following six phytogeographical regions: (a) Bohemian thermophyticum, (b) Pannonian thermophyticum, (c) Bohemian-Moravian mesophyticum, (d) Carpathian mesophyticum, (e) Bohemian-Moravian oreophyticum and (f) Carpathian oreophyticum (Fig. 5b).

Thermophyticum occurs in two separate areas – Bohemian and Pannonian. Bohemian thermophyticum is an isolated area of thermophilous vegetation in the northern half of Bohemia. It is a belt that stretches from areas lying in a rain shadow of the Ore Mountains in the north-western part of the Czech Republic, across Prague (the capital city of the Czech Republic) and reaching the city of Hradec Králové in the north-eastern part of the Bohemian region of the Czech Republic. On the other hand, Pannonian thermophyticum is located in southern part of the Moravian region and is connected to the forest-steppe area concentrated in the Pannonian Basin^42^ which lies partly in Slovakia and mainly in Hungary. With a bit of imagination, Pannonian thermophyticum forms a right triangle with upper peak near the city of Uničov, right peak near to Valašské Meziříčí in the south-eastern part, near to the borders with Slovakia, and left peak around the city of Znojmo, where it touches the Podyjí National park in the border with Austria.

Both Mesophyticum and Oreophyticum are divided according to their relationships with major mountain systems, floristic differences that reflect a similarity to Hercynian or Carpathian flora, respectively, and a gradient from oceanic to continental climate that increases from west to east. Most of the Mesophyticum that is connected with the Bohemian Massif is called Bohemian-Moravian mesophyticum^42^. It is the largest floristic region, as it occupies the vast majority of the Bohemian part of the Czech Republic and lies mainly in middle altitudes. The smaller part in eastern Moravia is recognized as Carpathian mesophyticum, which is associated with the West Carpathians. This phytogeographical region stretches in the middle altitudes and forms a belt along the borders with Austria towards the borders with Slovakia.

A similar approach as in the Mesophyticum division is used to distinguish Bohemian-Moravian oreophyticum and Carpathian oreophyticum^42^. Bohemian-Moravian oreophyticum occupies all high mountain ranges in the Bohemian region, lying mainly at the borders with other countries and partly some smaller inland areas with high altitudes as well. On the other hand, Carpathian mesophyticum is the smallest phytogeographical region in the Czech Republic. It is a small area with high altitudes in the south-eastern part of the country at the borders with Austria, surrounded by the phytogeographical region of Carpathian mesophyticum. Almost whole Carpathian oreophyticum is occupied by Nature Conservation Area of Beskydy, settlement there is sparse with several smaller villages, which helps to maintain and conserve its natural environment.

The dataset of orchid records used is based on the database of the Nature Conservation Agency of the Czech Republic. Classification and nomenclature of the taxa studied follows Danihelka *et al*.^43^, apart from *Dactylorhiza fuchsii* subsp. *carpatica* (Batoušek & Kreutz) Kreutz, which was also included in the species list, as it is an accepted taxon^72^.

Based on the literature^61,73–75^, the orchids were divided into nectariferous and nectarless species (Supplementary Table S1). For the genus *Epipactis*, the AHO-Bayern webpage^76^ was used. Nectariferous orchids are species that provide nectar to their pollinators as a reward for a pollen transfer^14^. On the other hand, nectarless orchids do not provide any kind of rewards (nectar in this case) for their pollinators and they use various kinds of deception to achieve pollination^18^.

Based on the coordinates of the orchid records, we obtained the altitude for each record with a 30-sec resolution (approximately 1 km^2^) from the WorldClim database^77^. As our main aim was to explore specific trends between orchid species density metrics and altitude, the altitudinal gradient in each phytogeographical region was divided into 100-m vertical intervals (i.e., 0–100 m, 101–200 m etc.), and the area (in km^2^) in each 100-m interval was estimated by counting the number of 30-sec grid cells in each altitudinal layer. An orchid was considered as present in a 100-m interval, only if it was recorded at least once in this interval. We did not assume that species have continuous distributions (as Grytnes & Vetaas^78^), because at a local scale, gaps in orchid distribution exist caused by unsuitable ecological conditions. After constructing the total matrix (in the form of presence/absence) for all the orchid taxa recorded in the Czech Republic, a series of orchid matrices was generated according to the traits studied. Specifically, for each orchid category (nectariferous, nectarless) the number of orchid taxa occurring in each vertical interval was calculated. This method does not take into account the degree of commonness or rarity of a species, but it is only based on the simple presence or absence of each species. In order to check for spatial differences within the Czech Republic, the territory of the country was divided according to the identified phytogeographical areas^42^ and the pre-described process was repeated separately for each of these areas.

Orchid species density, *D*, at each altitudinal interval was calculated using the formula:

$$D=S/log(A+1),$$

where S is number of orchid species recorded in each vertical interval and A is area of each vertical interval.

The relationships for the 6 phytogeographical regions was explored using a hierarchical cluster analysis applying the unweighted pair group method with an arithmetic mean (UPGMA) using Bray-Curtis similarity of square-root transformed occurrence data. The occurrence data matrix (69 orchids × 6 phytogeographical areas) was composed of the number of 30-sec grids where each orchid has been recorded in each phytogeographical area. The statistical significance of the identified clusters was calculated using a similarity profile analysis (SIMPROF^79,80^). Similarity profile analysis allows us to identify, whether the resulting groups (clusters) are significantly different from each other based on a given p-value (e.g. p <0.01 or p <0.05). This technique is a permutation test of the null hypothesis that phytogeographical areas do not form distinct groups.

An important attribute that is often used to test biogeographical theories is niche breadth or species tolerance. Niche breadth reflects the amplitude of the ecological conditions (e.g. climatic and habitat conditions) recorded where the species being studied occurs, which can include a variety of organisms. Here, we explore the trends between climatic niche breadth and altitude for the orchid groups studied for each phytogeographical region. As a measure of niche breadth, a species specialization index (SSI), calculated for each orchid was used^58^. Specifically, in the first step, we calculated species tolerance (%) using an outlying mean index analysis^81^ and the species specialization index (SSI) for each species was calculated using the following formula, according to the methodology described by Tsiftsis *et al*.^58^:

$$SSI=1-{T}_{i}/{T}_{max}$$

where *T*_*i*_ is the tolerance (%) of *i*_*th*_ species, and *T*_*max*_ is the maximum value of species tolerance (%) recorded.

It should be noted that low values of species tolerance imply that a species is distributed across habitats with a limited range of conditions (specialist species), whereas high values mean that a species is distributed across habitats with widely varying environmental conditions (generalist spcies)^81^. Contrary to the species tolerance values, SSI values range from 0 to 1, and species with low values indicate generalist species whereas large values indicate specialist species. This process was followed for each phytogeographical region by taking into account the orchids occurring in each area, as well as the 19 bioclimatic variables and the altitude of each area. After calculating the SSI values for each species in each area, we calculated the sum of the SSI values for each 100-m vertical interval on the basis of the species of each group recorded in a specific interval. As a final step, the mean SSI values were calculated by dividing the sum of the values by the number of orchids of each group recorded in each vertical interval.

In order to explore the associations between orchid species density and mean SSI with altitude, the data were analysed using regressions. As we did not have any *a priori* hypothesis about the functions describing the shape of the dependences studied, polynomial regressions were used. We first used third-degree polynomials and always tested the significance of the cubic terms, in order to determine, whether a second-degree or a linear regression would not be sufficient for fitting the data. In cases where neither cubic, nor quadratic terms were significant, we used linear regressions^9^.

The non-parametric Mann-Whitney U test was used to investigate (a) whether the number of orchid species is statistically different between the two orchid groups in the 10 × 10 grid cells of the total area of the Czech Republic, (b) whether the orchids in each group had a broader or coarser distribution on the basis of 10 × 10 grid cells or 30-sec grid cells in the whole territory of the Czech Republic, and (c) whether the two groups differed in the six phytogeographical regions, using also the 30-sec grid cells as a measurement unit.

All analyses were performed in R version 3.5.2 (R Foundation for Statistical Computing), whereas variable extraction was made using ArcGIS 10.1^82^.

## Supplementary information

Supplementary information.

## Data Availability

The datasets generated during and/or analysed during the current study are available in the Nature Conservation Agency of the Czech Republic repository, https://portal.nature.cz/publik_syst/ctihtmlpage.php?what = 2745&nabidka = rozbalitNadmodul&nadmodulID = 81.
