# Supplementary material for: Pollination Mechanisms are Driving Orchid Distribution in Space
Source: Sci Rep. 2020 Jan 21;10:850. doi: 10.1038/s41598-020-57871-5 (PMC6972782; doi:10.1038/s41598-020-57871-5)
Supplement: Supplementary file 1 — Supplementary information. [file 41598_2020_57871_MOESM1_ESM.pdf]

POLLINATION MECHANISMS ARE DRIVING ORCHID DISTRIBUTION IN SPACE

Štípková Zuzana*, Tsiftsis Spyros, Kindlmann Pavel

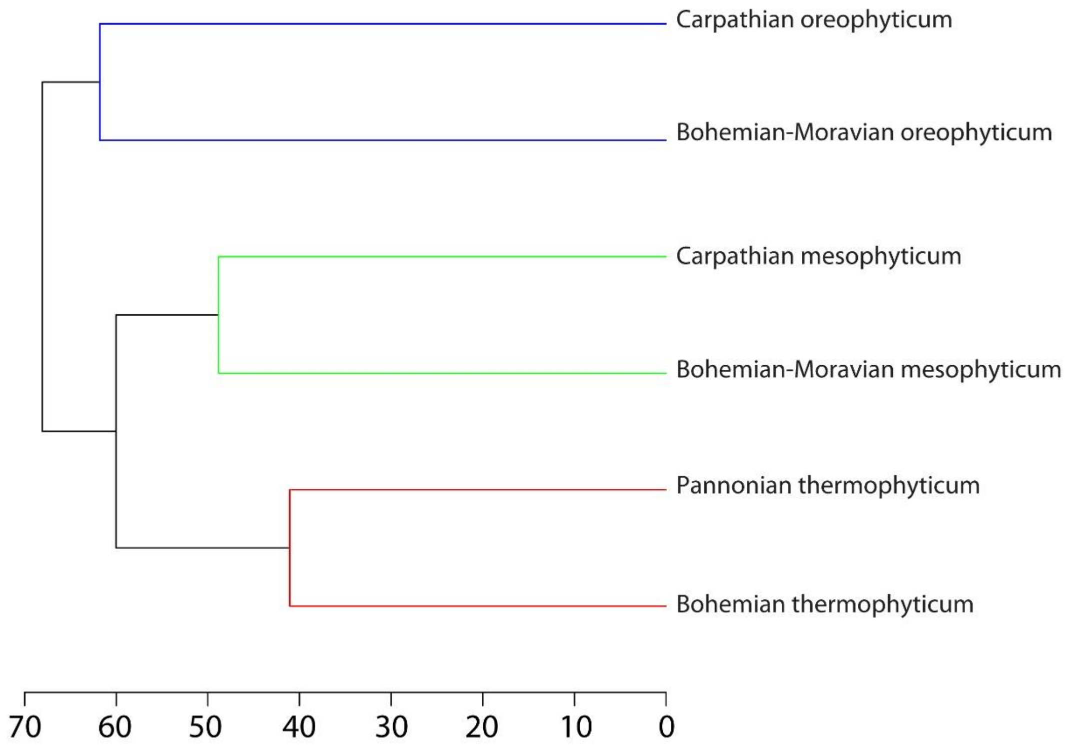

Supplementary Fig. S1 Hierarchical cluster analysis of the distribution of orchids using the SIMPROF test. Groups were formed according to similar altitudinal demands of typical flora and vegetation.

Supplementary Table **S1**: The list of nectariferous and nectarless species used in the analyses.

**Nectariferous species**	**Nectarless species**
Anacamptis coriophora subsp. *coriophora*	*Anacamptis morio*
Corallorhiza trifida	*Anacamptis palustris* subsp. *palustris*
Dactylorhiza viridis	*Anacamptis pyramidalis*
Epipactis albensis	*Cephalanthera damasonium*
Epipactis atrorubens	*Cephalanthera longifolia*
Epipactis futakii	*Cephalanthera rubra*
Epipactis greuteri	*Cypripedium calceolus*
Epipactis helleborine subsp. *helleborine*	*Dactylorhiza bohemica*
Epipactis helleborine subsp. *orbicularis*	*Dactylorhiza curvifolia*
Epipactis leptochila subsp. *leptochila*	*Dactylorhiza fuchsii* subsp. *fuchsii*
Epipactis leptochila subsp. *neglecta*	*Dactylorhiza fuchsii* subsp. *carpatica*
Epipactis leutei	*Dactylorhiza fuchsii* subsp. *sooana*
Epipactis microphylla	*Dactylorhiza incarnata* subsp. *incarnata*
Epipactis moravica	*Dactylorhiza incarnata* subsp. *serotina*
Epipactis muelleri	*Dactylorhiza maculata* subsp. *maculata*
Epipactis palustris	*Dactylorhiza maculata* subsp. *transsilvanica*
Epipactis pontica	*Dactylorhiza majalis* subsp. *majalis*
Epipactis pseudopurpurata	*Dactylorhiza majalis* subsp. *turfosa*
Epipactis purpurata	*Dactylorhiza sambucina*
Epipactis tallosii	*Dactylorhiza traunsteineri*
Epipactis voethii	*Himantoglossum adriaticum*
Epipogium aphyllum	*Liparis loeselii*
Goodyera repens	*Neotinea tridentata*

Gymnadenia conopsea	*Neotinea ustulata*
Gymnadenia densiflora	*Ophrys apifera*
Gymnadenia odoratissima	*Ophrys holoserica* subsp. *holubyana*
Hammarbya paludosa	*Ophrys insectifera*
Herminium monorchis	*Orchis mascula*
Limodorum abortivum	*Orchis militaris*
Malaxis monophyllos	*Orchis pallens*
Neottia cordata	*Orchis purpurea*
Neottia nidus-avis	*Traunsteinera globosa*
Neottia ovata	
Platanthera bifolia	
Platanthera chlorantha	
Pseudorchis albida	
Spiranthes spiralis	

Supplementary Table **S2**. Comparisons of the distributions of nectariferous and nectarless orchids in 6 phytogeographical areas in the Czech Republic based on Mann-Whitney U tests.

**Phytogeographical area**	**Most broadly distributed orchid group**	**P value**
Bohemian thermophyticum	Nectarless	P = 0.344
Bohemian-Moravian mesophyticum	Nectariferous	P = 0.425
Bohemian-Moravian oreophyticum	Nectariferous	P < 0.05
Pannonian thermophyticum	Nectarless	P = 0.197
Carpathian mesophyticum	Nectariferous	P < 0.05
Carpathian oreophyticum	Nectariferous	P = 0.274

Supplementary Table **S3**. Summary statistics of the polynomial regressions of orchids' diversity along the altitudinal gradient.

Biogeographical region	Pollination mechanism	R^2	P value
Bohemian-Moravian oreophyticum	Nectariferous	0.84(c)	P<0.001
Bohemian-Moravian oreophyticum	Nectarless	0.62(c)	P<0.01
Bohemian thermophyticum	Nectariferous	0.77(b)	P<0.05
Bohemian thermophyticum	Nectarless	0.72(b)	P<0.05
Bohemian-Moravian mesophyticum	Nectariferous	0.11(a)	NS
Bohemian-Moravian mesophyticum	Nectarless	0.62(a)	P<0.01
Carpathian mesophyticum	Nectariferous	0.86(b)	P<0.001
Carpathian mesophyticum	Nectarless	0.72(b)	P<0.01
Carpathian oreophyticum	Nectariferous	0.73(c)	P<0.01
Carpathian oreophyticum	Nectarless	0.87(c)	P<0.001
Pannonian thermophyticum	Nectariferous	0.72(a)	P<0.05
Pannonian thermophyticum	Nectarless	0.99(b)	P<0.001
(a): 1st order polynomial regression; (b): 2nd order polynomial regression; (c): 3rd order polynomial regression			

Supplementary Table **S4**: Summary statistics of the polynomial regressions of the mean SSI values along the altitudinal gradient.

Biogeographical region	Pollination mechanism	R^2	P value

Bohemian-Moravian oreophyticum	Nectariferous	0.89(c)	P<0.001
Bohemian-Moravian oreophyticum	Nectarless	0.81(b)	P<0.001
Bohemian thermophyticum	Nectariferous	0.59(b)	NS
Bohemian thermophyticum	Nectarless	0.93(c)	P<0.01
Bohemian-Moravian mesophyticum	Nectariferous	0.65(c)	P<0.01
Bohemian-Moravian mesophyticum	Nectarless	0.88(b)	P<0.001
Carpathian mesophyticum	Nectariferous	0.93(b)	P<0.001
Carpathian mesophyticum	Nectarless	0.55(b)	P<0.05
Carpathian oreophyticum	Nectariferous	0.57(c)	P<0.05
Carpathian oreophyticum	Nectarless	0.51(b)	P<0.05
Pannonian thermophyticum	Nectariferous	0.36(b)	NS
Pannonian thermophyticum	Nectarless	0.31(a)	NS
(a): 1st order polynomial regression; (b): 2nd order polynomial regression; (c): 3rd order polynomial regression			
